# Trends in prevalence, incidence, health system use and cost by persons with dementia in Ontario from 2004 and 2013: a population-based study

**DOI:** 10.23889/ijpds.v1i1.321

**Published:** 2017-04-19

**Authors:** Susan Bronskill, Jun Guan, Marian Vermeulen, Erika Yates, Ryan Ng, Xuesong Wang, Jennifer Walker, Colleen Maxwell

**Affiliations:** 1 Institute for Clinical Evaluative Sciences (ICES)

## Objectives

Efforts to enable persons with dementia to remain at home longer, and to reduce use of costly acute care resources, are at the forefront of policy agendas internationally. Foundational to planning appropriate health system supports is the ongoing, comparable and accurate estimation of the prevalence and incidence of dementia across regions, as well as associated patterns of health services use and cost. Our objective was to explore emerging approaches to using population data in dementia research and demonstrate the policy contribution of the resulting new knowledge.

## Approach

Using population-based health administrative data and an algorithm that was validated using electronic medical records, we developed a series of repeated, cross-sectional cohort studies to examine trends in dementia prevalence, incidence and publicly-funded health service use and costs between 2004/05 and 2013/14 among adults aged 65 years and older in Ontario, Canada. Trends in yearly rates of health service use were assessed using regression models for serially correlated data and compared to a 1:1 matched control group based on age, sex, geographic region and comorbidity level.

## Results

Over time, age- and sex-adjusted prevalence of dementia increased by 18.2% (from 63.0 to 74.5 per 1,000 persons; p-value < 0.001) and age- and sex-adjusted incidence decreased slightly (from 18.2 to 17.0 per 1,000 persons; p-value = 0.05). Community-dwelling persons with dementia were more likely than matched controls to be placed in long-term care (11.8% vs. 1.5% in 2013; p<0.001) and use home care (45.8% vs 23.2%; p<0.001) but equally likely to visit family physicians (93.9% vs. 94.8% in 2013) and specialists (87.1% vs. 89.4%). Median costs associated with one year of health system use were $19,468 (interquartile range (IQR) $4,490 to $47,726) for prevalent cases in 2012/13 and $16,549 (IQR $5,070 to $47,899) for incident cases. Long-term care and hospital care accounted for the largest portion of total costs in both groups.

## Conclusions

We observed a weak positive association between LDL-C and AF in this study. This is the first Mendelian randomization approach that focuses on this relationship. More Mendelian randomization studies should be performed in order to identify the causal effect of LDL-C to AF. The use of electronic health records will facilitate the conduction of similar studies.

